# Topical pharmacotherapy for ocular surface squamous neoplasia: systematic review and meta-analysis

**DOI:** 10.1038/s41598-022-18545-6

**Published:** 2022-08-20

**Authors:** Kincső Kozma, Zsuzsa Réka Dömötör, Adrienne Csutak, László Szabó, Péter Hegyi, Bálint Erőss, Zsuzsanna Helyes, Zsolt Molnár, Fanni Dembrovszky, Eszter Szalai

**Affiliations:** 1 Department of Ophthalmology, University of Pecs, Rakoczi u. 2, 7623 Pecs, Hungary; 2 Department of Ophthalmology, Faculty of Medicine, University of Debrecen, Nagyerdei krt. 98, 4032 Debrecen, Hungary; 3 Institute for Translational Medicine, Szentágothai Research Centre, Medical School, University of Pécs, Pécs, Hungary; 4 Centre for Translational Medicine, Semmelweis University, Budapest, Hungary; 5 Division of Pancreatic Diseases, Heart and Vascular Center, Semmelweis University, Budapest, Hungary; 6 Department of Pharmacology and Pharmacotherapy, Medical School, University of Pécs, 7624 Pécs, Hungary; 7 János Szentágothai Research Centre, University of Pécs, 7624 Pécs, Hungary; 8 Department of Anesthesiology and Intensive Therapy, Poznan University of Medical Sciences, Poznan, Poland; 9 Department of Anaesthesiology and Intensive Therapy, Semmelweis University, Faculty of Medicine, Budapest, Hungary

**Keywords:** Cancer, Oncology

## Abstract

Ocular surface squamous neoplasia (OSSN) has different treatment modalities. Although surgical excision has been the gold standard therapeutic option, topical pharmacotherapy agents such as 5-fluorouracil (5-FU), interferon alfa-2b (IFN) and mitomycin-C (MMC) are also commonly used. The protocol was registered (CRD42021224961). Comprehensive literature research was carried out to compare topical pharmacotherapy (5-FU or IFN or MMC) to surgical excision regarding clinical success (tumor resolution), recurrence and complications in patients undergoing treatment for OSSN. From 7859 records, 7 articles were included in the qualitative and 4 in the quantitative synthesis. The outcomes of surgical excision and topical pharmacotherapy were comparable in the included articles. There were no significant differences between surgical excision and topical pharmacotherapy regarding the clinical success [odds ratio (OR): 0.785; confidence interval (CI): 0.130–4.736, P = 0.792)] and tumor recurrence (OR: 0.746; CI: 0.213–2.609; P = 0.646). The most common side effect of the different therapeutic options was dry eye. The highest rate of dry eye symptoms was reported after surgical excision (in 59%). Topical pharmacotherapy with all the 3 agents is as effective and well-tolerable as surgical excision in terms of tumor resolution, recurrence rate and side effects in all OSSN patients suggesting similar long-term clinical benefits.

## Introduction

Ocular surface squamous neoplasia (OSSN) is an uncommon disease, which includes a spectrum of slowly progressive benign, premalignant and malignant epithelial lesions of the conjunctiva and cornea^1^. Although most of these neoplasias are rare with an incidence of 0.1–35 cases/1,000,000 people, they might have a considerable impact on the patients’ morbidity and mortality^2^. Risk factors include ultraviolet light and sun exposure, vitamin A deficiency, trauma, inflammation, xeroderma pigmentosum (XP), type 1 and 2 of human immunodeficiency virus (HIV-1, HIV-2), human papilloma virus (HPV) infections and other immunosuppressive conditions^3–8^.

The clinical appearance of OSSN is characterized by thickening of the squamous epithelium. The lesion can be flat or elevated, focal or diffuse, isolated or multifocal, and may have an atypical vascular pattern and exhibit surface keratinization^9^. The adjacent conjunctiva can also be affected with prominent feeder vessels. Since OSSN lesions frequently arise from the mitotically active limbal stem cells, patients typically present with a gelatinous, papilliform or leukoplakic soft lesion mostly located in the juxta-limbal part of the cornea^10^. These include conjunctival/corneal intraepithelial neoplasia when the basement membrane remains intact and epithelial cells in the basal germinative layer are replaced by dysplastic cells. In carcinoma in situ, the basement membrane is still intact but full thickness of the epithelium is replaced by dysplastic cells. In invasive squamous cell carcinoma, the malignant epithelial cells penetrate the basement membrane into the stroma^1^. Some studies suggest that more advanced disease is related to higher recurrence rate^11^, however others found no increased risk of recurrence related to higher initial TNM category^12,13^.

Diagnosis based on clinical presentation could be challenging due to the variable clinical appearance of the lesions. The differential diagnosis includes pterygium, pinguecula, conjunctival cyst, hemangioma, pyogenic granuloma, actinic keratosis, benign intraepithelial dyskeratosis, xerophthalmia and melanocytic lesions^14^. The gold standard diagnostic technique is excisional biopsy using a “no-touch technique” described by Shields and colleagues^15^. However, non-invasive imaging modalities such as anterior segment optical coherence tomography (AS-OCT) offer a diagnostic alternative to biopsy by providing high-resolution cross-sectional images of the ocular surface. Kieval et al. found that using an epithelial thickness cut-off of 142 µm had a sensitivity of 94% and a specificity of 100% for differentiating between OSSN and pterygia^16^, whereas a study by Nanji et al. reported that a thickness of 120 µm had a sensitivity and specificity of 100%^17^. The main limitation of AS-OCT is that it cannot specify the absence or the presence of tumor invasion beyond the basement membrane^18^. In vivo confocal microscopy (IVCM) is a real time virtual biopsy providing tissues' architectural information and low-magnification cytology that allows non-invasive presumptive diagnosis of ocular surface lesions. The sensitivity and specificity of IVCM for distinguishing OSSN from benign conjunctival lesions in East Africa were 38.5% and 66.7%, respectively^19^. Limitations of IVCM include diagnosing deep extension is often challenging because optical sections are recorded in a coronal plane and optically dense tumor growth of the epithelium extending deep into the stroma may degrade the image quality.

Ultrasound biomicroscopy is most applicable in evaluating intraocular tumor extension and metastatic spread^20^. It may be an adjunctive imaging modality to high-resolution AS-OCT^21^. Non-invasive anterior segment imaging modalities are useful in the diagnostic process and for monitoring the response to topical pharmacotherapy and early identification of recurrence.

There is considerable overlap in clinical features of OSSN spectrum diseases and amelanotic melanocytic ocular surface lesions. OSSN may have a variable amount of pigmentation, although pigmented OSSN is a rare entity in the Caucasian population^22^. The characteristic clinical appearance and AS-OCT features may help to differentiate between pigmented OSSN from conjunctival/corneal melanocytic tumors^23^. As opposed to OSSN, melanoma demonstrates a hyper-reflective subepithelial mass with mild epithelial thickening^17^. Kaliki et al. reported a complete resolution of tumor-related pigmentation and 100% of tumor resolution in patients with pigmented OSSN treated with a combination of subconjunctival and topical interferon alfa-2b^23^.

Traditionally ocular surface malignancies have been treated with surgical excision with adjuvant cryotherapy (double freeze thaw technique)^15^. However, recurrence rates after surgical excision can be high and extended or repeated surgeries may lead to scarring of the ocular surface and/or limbal stem cell deficiencies^24^, thus non-surgical treatment options have been used increasingly.

Topical pharmacotherapeutical agents applied for OSSN include 5-fluorouracil (5-FU), interferon alfa-2b (IFN) and mitomycin-C (MMC). These can be applied solely or in combination with surgical excision^25^. 5-FU is a pyrimidine analog, it promotes apoptosis in cells in a cell cycle dependent fashion through several mechanisms^26^. IFN is an inducible glycoprotein produced by human immune cells, which has immunomodulatory, antiproliferative, and angiogenesis inhibitory effects. It also has antiviral and antitumoral actions^27^. MMC is an alkylating agent derived from *Streptomyces* *caespitosus*, which induces deoxyribonucleic acid (DNA) direct damage in rapidly proliferating cells^28^.

The aim of this meta-analysis was to compare topical pharmacotherapy (5-FU or IFN or MMC) to surgical excision regarding clinical success, recurrence and complication in patients undergoing treatment for ocular surface squamous neoplasia.

## Methods

This meta-analysis was performed using the population intervention-control-outcomes (PICO) method. Retrospective comparative studies were selected where patients with clinically and/or histopathologically diagnosed OSSN (P) were treated with topical pharmacotherapy (either with IFN or MMC or 5FU) or surgical excision and at least two treatment modalities were compared (I and C). Clinical success (tumor resolution), recurrence, complications including pain, hyperaemia, dry eye, keratopathy with or without limbal stem cell deficiency, systemic side effects were analyzed as the outcomes of different treatment options (O).

The meta-analysis was reported in accordance with the Preferred Reporting Items for Systematic Review (PRISMA) statement^29^, and it was registered in advance in the PROSPERO database (registration number: CRD42021224961).

### Search strategy, inclusion and exclusion criteria

The electronic databases of Medline, Embase and Cochrane Central Register of Controlled Trials were systematically searched up to 6th March 2022 for relevant studies without language restrictions. The search included the following keywords: (ocular surface squamous neoplasia) OR (conjunctiva* carcinoma) OR (conjunctiva* squamous cell carcinoma) OR (ocular surface squamous cell carcinoma) OR (cornea* carcinoma) OR (conjunctiva* neoplasia) OR (cornea* neoplasia) OR (cornea neoplasia) OR (conjunctiva carcinoma) OR (conjunctiva squamous cell carcinoma) OR (cornea carcinoma) OR (conjunctiva neoplasia)) AND (surgery OR topical OR local OR surgical OR excision OR mitomycin OR interferon OR fluorouracil OR '5 fu' OR '5 fluorouracil’. No search filters were applied.

Articles were included if they provided data on at least two of the treatment modalities on patients suffering from OSSN reporting the outcomes mentioned above. Retrospective and prospective comparative studies were selected. Case reports, duplicates, and results from non-human trials were excluded.

### Selection process and data extraction

The publications were processed by the EndNote X7.4 software (Clarivate Analytics, Philadelphia, PA, USA). Titles, abstracts and full texts of studies were retrieved using the search strategy and were screened independently by two review authors (KK and ZRD) to identify studies that potentially meet the inclusion criteria. The full texts of these potentially eligible studies were retrieved and independently assessed for eligibility by two review team members. Any disagreement between them over the eligibility of particular studies was resolved through discussion with a third reviewer. From the included articles we extracted demographic data, data related to the outcomes.

At each level of selection Cohen’s kappa coefficient (κ) was calculated to evaluate the interrater reliability. κ values ≤ 0 were considered as no agreement and 0.01–0.20 as none to slight, 0.21–0.40 as fair, 0.41–0.60 as moderate, 0.61–0.80 as substantial, and 0.81–1.00 as almost perfect agreement^30^.

Numerical data were extracted in an Excel (Office 365, Microsoft, Redmond, WA, USA) spreadsheet designed for this purpose. The investigators (KK and ZRD) extracted the number of subjects, methods of treatment, clinical success, recurrence, complications: pain, hyperaemia, dry eye, keratopathy with or without limbal stem cell deficiency, systemic side effects from each publication independently, and then validated these data. Disagreements were discussed and resolved by consensus in plenum.

### Statistical methods, data synthesis

Pairwise comparison between the treatment modalities (surgical excision, 5-FU, IFN, MMC topical pharmacotherapy) were performed with the outcomes of recurrence and clinical success. For each binary outcome, the odds ratio (OR) was calculated for each study and these ORs were pooled with the random effect model (DerSimonian and Laird estimation)^31^. The ORs and the pooled estimates were displayed on forest plots with the corresponding 95% confidence interval, weights, and p-value. Statistical heterogeneity was analyzed using the I^2^ statistic and the χ^2^ test to gain probability-values; P < 0.1 is defined to indicate significant heterogeneity. The I^2^ corresponds to the percentage of total variability across studies because of heterogeneity. Based on Cochrane’s handbook I^2^ values of 30–60% are interpreted as moderate, 50–90% as substantial and 75–100% as considerable heterogeneity^32^. Where mean and standard deviation is not reported, it was estimated from median, IQR, minimum and maximum values by using the method of Xiang Wan^33^. Funnel plots were created to visually detect the presence of publication bias.

### Quality assessment of the studies included

Two authors (KK and ZRD) independently performed the risk of bias assessment for every examined outcome according to the Cochrane recommendation using the ROBINS-I tool: Risk Of Bias In Non-randomized Studies—of Interventions. Disagreements between the review authors over the risk of bias in particular studies were resolved by discussion, with involvement of a third review author where necessary.

### Assessment of the grade of evidence

The GRADE (Grading of Recommendations Assessment, Development, and Evaluation) system was used to assess the strength of recommendation and quality of evidence of our results. We classified our results into four levels: high, moderate, low, and very low certainty of evidence.

### Ethics approval and consent to participate

Not required as data is not individualized and primary data was not collected. Not required as data is not individualized and primary data was not collected.

### Consent for publication

The corresponding author accepts responsibility for releasing this material on behalf of all co-authors.

## Results

### Results of the selection process

**Figure 1:**
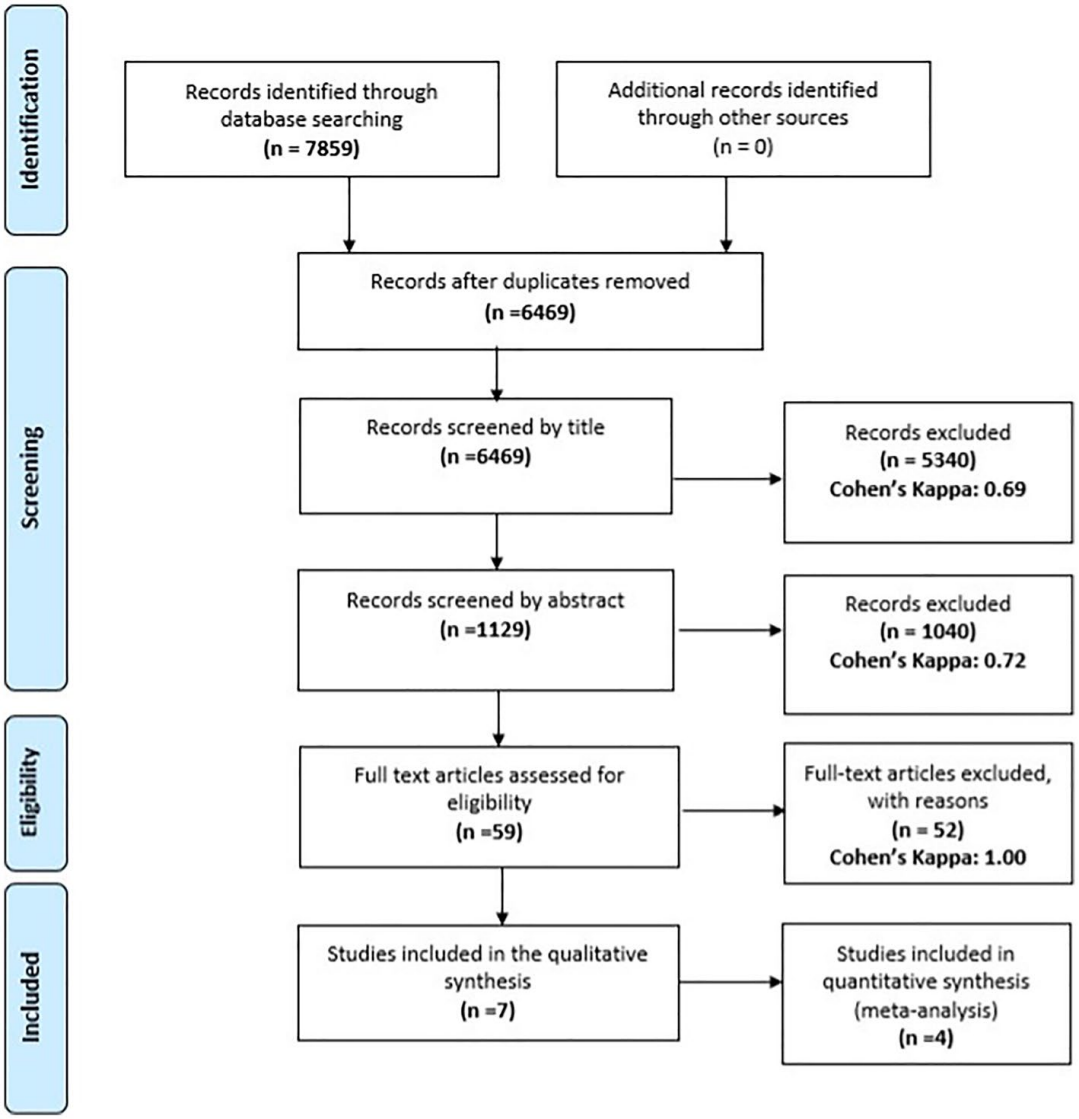
PRISMA (preferred reporting items for systematic reviews and meta-analyses) flowchart showing the different phases of this systematic review and meta-analysis.

We identified 7859 articles in the Medline, Embase and Cochrane Central Register of Controlled Trials databases. Once duplicates had been removed, 6469 studies were screened by title, followed by 1129 studies screened by abstract, and 59 full text articles were assessed for eligibility. At the end of the selection process 7 articles were eligible. The inter-rater reliability was rated as substantial or perfect at all steps of the selection. The process of the selection is summarized in Fig. 1.

### Characteristics of the studies included

Four eligible articles were included in the quantitative synthesis^12,13,34,35^ and 3 more articles in the qualitative synthesis of this meta-analysis^36–38^. They included 4 cohort studies with 159 patients with OSSN in the qualitative synthesis and 3 more studies with 159 patients in the quantitative synthesis; in total 318 patients were included in the review. The risk of bias varied slightly across the studies (Supplemental Multimedia Component 1–18). The characteristics of the eligible studies, demography of patients, treatment methodology, definition of clinical success, recurrence and duration of follow-up are shown in Tables 1, 2, 3. In most studies surgical excision was performed with “no-touch” technique, including conjunctival incision, with a 3–4 mm surgical margins. In two studies double freeze–thaw cryotherapy was performed along the conjunctival margins^12,13^. In one study after the excision of the lesion, a thin scleral flap beneath the tumor was removed^34^. In terms of topical pharmacotherapy one drop of each agent was applied for four times a day, with a variable length of treatment. The usual concentration of IFN eye drop was 1 million IU/mL. In some studies, intralesional subconjunctival IFN was also administered at a concentration of 3 million IU/0.5 mL (Table 3). Topical 5-FU was administered at a concentration of 1% four times a day for 1 week. MMC was applied in a week on/week off regime in most studies at a concentration of 0.02–0.04%. The treatment cycle characteristics and exact doses varied between studies (Table 3).

**Table 1:** Characteristics of studies included in the quantitative synthesis. *ND* no data, *EXC* surgical excision.

Publication data		GR 1	GR 2	Design	Demography				Total number of patients	Definition of clinical success	Definition of recurrence	Follow up (months)
First author	Year of publication				Country, Institution	Age (years) Group Surgical Excision		Female ratio % Group 1				
						Mean	Standard deviation					
Sturges et al.^34^	2008	EXC	IFN	Retrospective, comparative, interventional study	USA—Oklahoma, Dean McGee Eye Institute	65.20	14.20	10.22	29	Clinical resolution of the tumor, disease- free follow up	ND	Mean: 35.6
Tanabe et al.^35^	2013	EXC	MMC	Retrospective	Japan -Kyushu University Graduate School of Medicine	69.45	8.21	30	10	Not specified	ND	12–120
Nanji et al.^17^	2014	EXC	IFN	Retrospective, matched case–control study	USA—University of Miami, Bascom Palmer Eye Institute	64	14.5	47	98	Clinical resolution of the tumor, disease- free follow up	Reappearance of the lesion in the same/similar location, after complete resolution	IFN: 21 EXC: 24
Polski et al.^13^	2019	EXC	IFN, MMC, 5-FU	Retrospective, comparative study	USA—University of Southern California Roski Eye Institute	70.25	10.95	ND	22	Clinical resolution of the tumor, disease- free follow up	Reappearance of the lesion after complete resolution	1.5–6.5

**Table 2:** Characteristics of studies included only in the qualitative synthesis ND: no data.

Publication data		GR 1	GR 2	Design	Demography				No. of patients	Definition of clinical success	Definition of recurrence	Mean follow up (months)
First author	Year of publication				Country, institution	Age (years) group surgical excision		Female ratio % Group 1				
						Mean	Standard deviation					
Chaugule et al.^36^	2017	IFN	5-FU	Retrospective, interventional series	USA—The New York Eye Cancer Center	ND	ND	ND	6	Clinical resolution of the tumor, defined on slit-lamp examination	Reappearance of the lesion at a similar location, after complete resolution	IFN: 8.8; 5-FU: 18
Kusumesh et al.^37^	2017	IFN	MMC	Retrospective, comparative study	Patna, India—Cornea Services, Regional Institute of Ophthalmology, Indira Gandhi Institute of Medical Sciences	59.5	14.55	ND	51	Total disappearance of lesions with clear visibility of underlying structures	Reappearance of a tumor at the same location or at any other location after complete resolution	IFN: 22.2 MMC: 23.6
Venkateswaran et al.^38^	2018	IFN	5-FU	Retrospective, comparative, interventional case series	USA—University of Miami, Bascom Palmer Eye Institute	67.5	11	ND	102	Disappearance of the lesion clinically and/or by AS-OCT	Reappearance of a tumor at the same/similar location or at any part of the ocular surface after complete resolution	5-FU: 15.8 IFN: 20.9

**Table 3:** Characteristics of treatments. UI/mL: International units per millilitre; subconj. inj.: subconjunctival injection; M: million; mL: millilitre.

Publication data	Intervention							
First author	Group 1				–			
	Intervention	No. of patients	Dose	Administration	Intervention	No. of patients	Dose	Administration
Sturges et al.^34^	Excision	14		Excision with 4 mm surgical margins and removal a thin scleral flap beneath the tumor	IFN	15	Solution 1 M IU/mL	4 × 1 drop/day
Tanabe et al.^35^	Excision	5		Excision with 3 mm surgical margins	MMC	5	0.04%	4 × 1 drop/day; 1 week on–1–2 weeks off
Nanji et al.^17^	Excision	49		Excision (4 mm margins) + freeze thaw cryotherapy (41 case), + intraoperative mitomycin C was (1 case), + sclerectomy (6 cases) Amniotic membrane (14 cases), conjunctival autograft in 1 case	IFN	49	drop: 3 M IU (in 0.5 mL); inj.:1 M IU/mL; drop + inj: 3 M IU/mL	4 × 1 drop/day
Chaugule et al.^36^	IFN	5	Intron A powder reconstituted to topical 1 M IU/mL	4 × 1 drop/day for 3 months	5-FU	1	Concentration of 1%	4 × 1 drop/day for 2 weeks
Kusumesh et al.^37^	IFN	26	1 mL of recombinant IFNa2b injection + 2 mL of 0.9% NaCl	4 × 1 drop/day	MMC	25	2 mg powder with 5 mL of 0.9% NaCl	4 × 1 drop/day, (0.4 mg/mL or 0.04%) week-on–week-off regimen
Venkateswaran et al.^38^	IFN	48	Concentration of 1 M IU/mL	4 × 1 drop/day, no pause	5-FU	54	Concentration of 1%	4 × 1 drop for 1 week on–3 weeks off
Polski et al.^13^	Excision	12		Excisional biopsy with no-touch technique + double freeze–thaw cryotherapy along conjunctival margins	IFN, MMC, 5-FU	10	5-FU—1%; MMC (0.02%); IFN drop (1 million IU/mL); IFN inj (3 million IU in 0.5 mL	5-FU 4 × 1 drop/day—(2 weeks on–2 weeks off × 2 courses), MMC 4 × 1 drop/day—(2 weeks on–2 weeks off × 2 courses), IFN 4 × 1 drop/day—(4–6 months continuously), and/or IFN-a2b subconj inj. 3–6 injections—weekly)

### Synthesis

#### Clinical success

**Figure 2:**
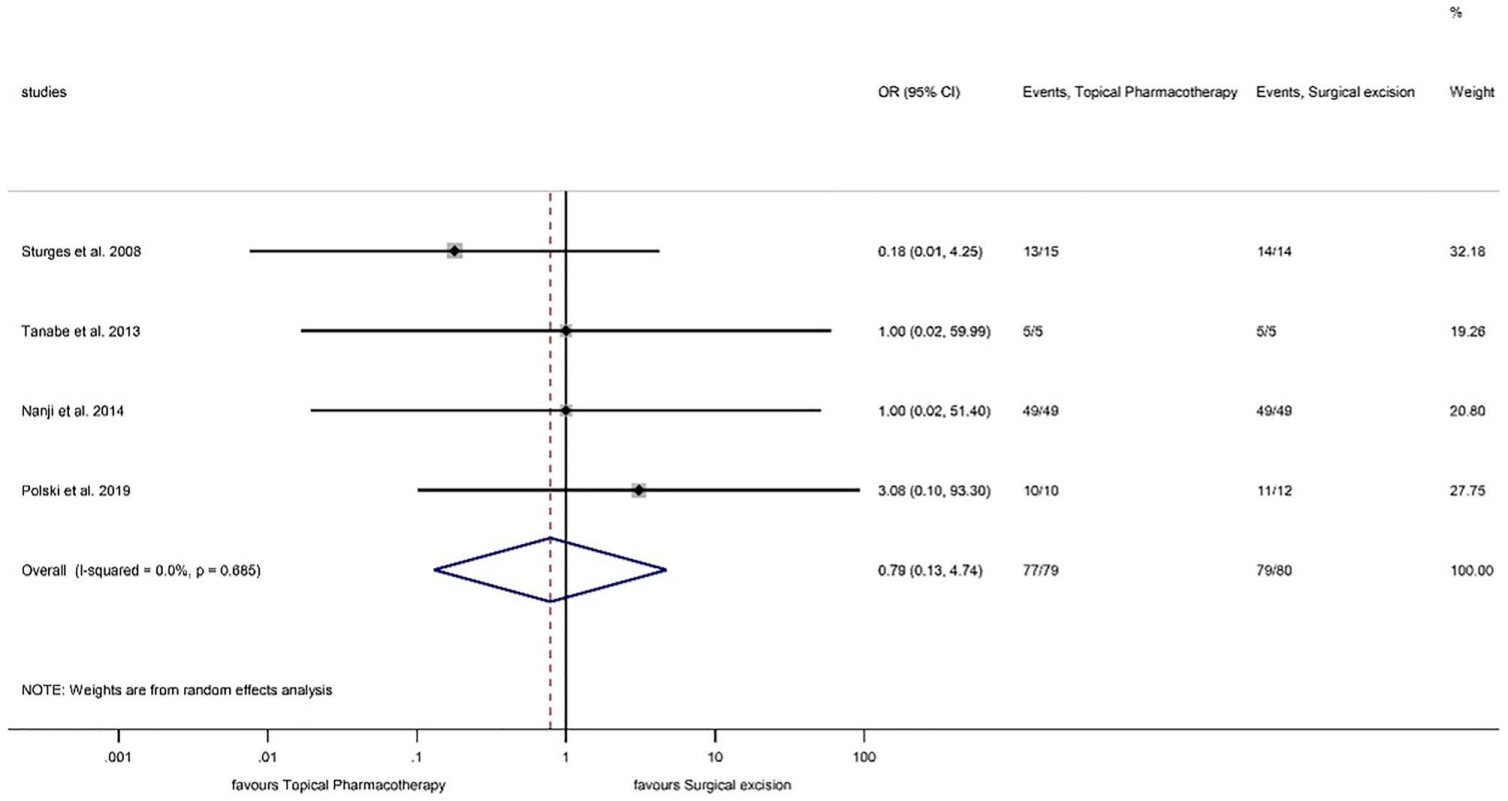
Forest plot of studies measuring clinical success of different treatment modalities of OSSN. The clinical success of each study and the 95% confidence intervals (CIs) are presented. The diamond at the bottom represents the overall clinical success for all studies.

**Figure 3:**
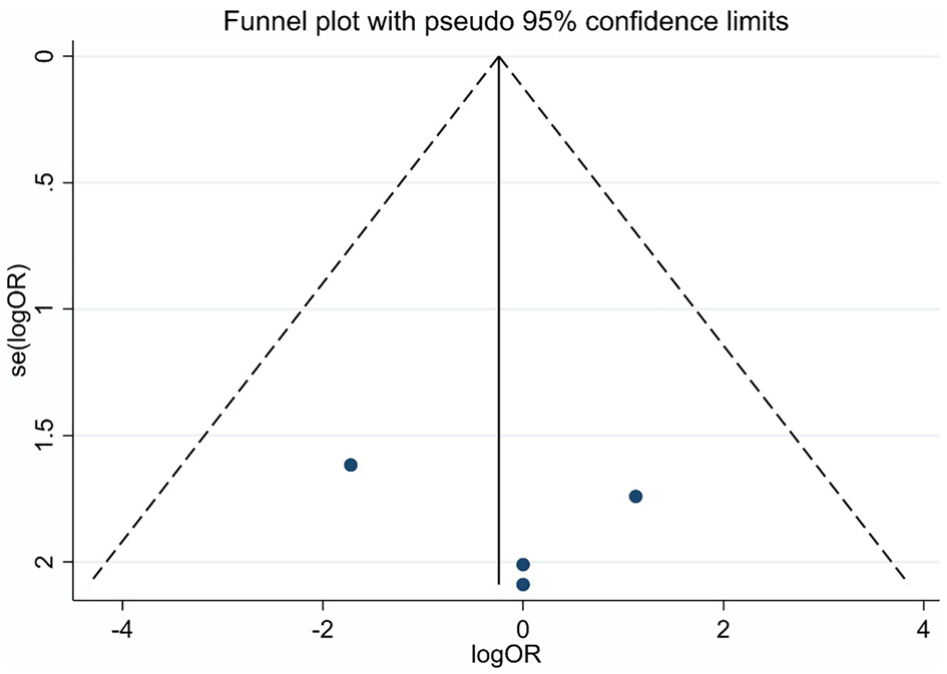
Funnel plot of studies assessing clinical success of different treatment modalities of OSSN.

Forest plot of the 4 studies^12,13,34,35^ indicated no significant difference between topical pharmacotherapy with any of the 3 agents and surgical excision regarding clinical success (OR: 0.785; CI: 0.130–4.736, P = 0.792) (Fig. 2). No detectable heterogeneity was observed among the studies (P = 0.685). Visual assessment of the funnel plot indicated that publication bias was not present (Fig. 3). Two studies compared IFN with 5-FU^36,38^. In the first study, 5 patients were treated with IFN and 1 patient with 5-FU, the tumor resolution was complete in all cases^36^. In the second study, 48 patients received IFN and 52 were treated with 5-FU, clinical success was 81.2% in the case of IFN and 96.2% in the case of 5-FU^38^. When IFN and MMC were compared, out of 26 IFN-treated patients, tumor resolution was complete in 23 cases; and out of 25 patients receiving MMC, 23 cases showed complete tumor resolution^37^.

#### Recurrence rate

**Figure 4:**
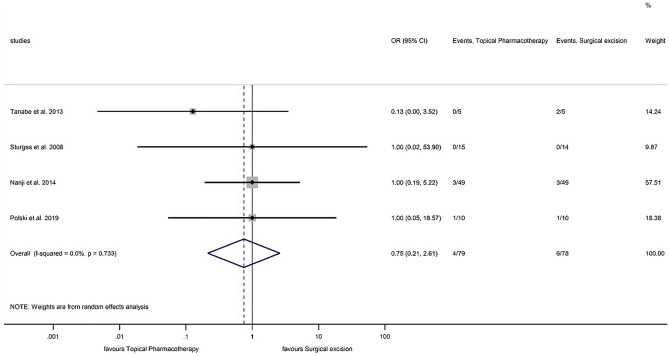
Forest plot of studies measuring tumor recurrence of different treatment modalities in OSSN. The recurrence rate of each study and the 95% confidence intervals (CIs) are presented. The diamond at the bottom represents the overall recurrence rate for all studies.

**Figure 5:**
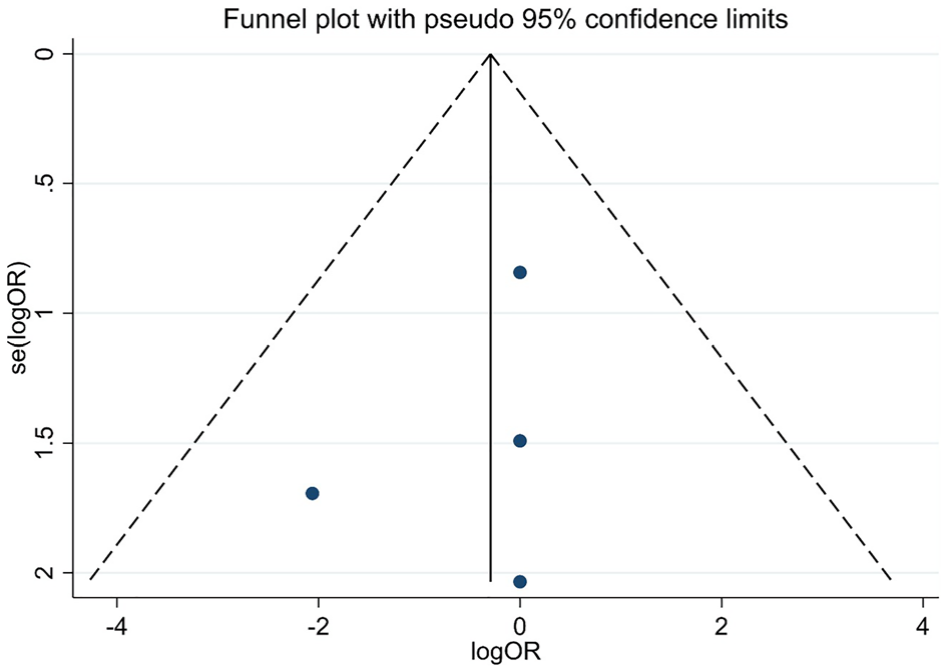
Funnel plot of studies assessing tumor recurrence of different treatment modalities in OSSN.

The forest plot does not indicate significant difference between topical pharmacotherapy and surgical excision regarding tumor recurrence (OR: 0.746; CI: 0.213–2.609; P = 0.646) (Fig. 4). No detectable heterogeneity was observed among the studies (P = 0.733). The funnel plot does not suggest any relevant publication bias (Fig. 5). Neither IFN (5 patients) nor 5-FU (1 patient) treatment resulted in recurrence during the follow-up period^36^. In another study, 5.1% and 11.1% recurrence occurred in IFN (48 patients) and 5-FU-treated patients (52 patients), respectively^38^. Furthermore, among 26 IFN and 25 MMC-treated patients, only one recurrence occurred after IFN treatment^37^.

#### Complications and side effects of different treatments

Descriptive analysis of complications including pain, hyperaemia, dry eye, keratopathy with or without limbal stem cell deficiency and systemic side effects (pain, hyperaemia, dry eye, keratopathy with or without limbal stem cell deficiency) was performed due to the limited data availability. Four studies were included in this analysis (Table 4). The most common side effect in all cases was dry eye. The highest rate of dry eye symptoms was reported after surgical excision (59% of the patients). The second most common side effect was conjunctival hyperaemia, its highest rate (44% of the patients) occurred after topical MMC. Pain was reported both after surgical excision and topical pharmacotherapy, but in a higher rate after surgical excision (40.8% after surgery). Keratopathy was reported after topical pharmacotherapy with MMC (12% of the patients) and 5-FU (9% of the patients), but there was no limbal stem cell deficiency in any of the included studies. Two studies reported systemic side effect rate of 12.9% after IFN, most commonly flu-like symptoms, such as fever, chills, myalgia and fatigue^12,13^.

**Table 4:** Complications and side effects of different treatment modalities.

Therapy	Surgical excision					
Side effect	Dry eye	Hyperaemia	Pain	Keratopathy	LSCD	Systemic side effects
Nr. of studies included	1	1	1	1	1	ND
Total nr. of patients	49	49	49	49	49	ND
Side effect rate (%)	59%	38.7%	40.8%	0%	0%	ND

Therapy	IFN					
Side effect	Dry eye	Hyperaemia	Pain	Keratopathy	LSCD	Systemic side effects
Nr. of studies included	3	4	2	3	3	2
Total nr. of patients	128	128	97	102	102	54
Side effect rate (%)	34.3%	17.1%	21.6%	0%	0%	12.9%

Therapy	5-FU					
Side effect	Dry eye	Hyperaemia	Pain	Keratopathy	LSCD	Systemic side effects
Nr. of studies included	2	2	1	2	2	ND
Total nr. of patients	55	55	54	55	55	ND
Side effect rate (%)	23.6%	21.8%	22.2%	9%	0%	ND

Therapy	MMC					
Side effect	Dry eye	Hyperaemia	Pain	Keratopathy	LSCD	Systemic side effects
Nr. of studies included	1	11	ND	1	ND	ND
Total nr. of patients	25	25	ND	25	ND	ND
Side effect rate (%)	36%	44%	ND	12%	ND	ND

*LSCD* limbal stem cell deficiency.

## Discussion

In this meta-analysis we compared the efficacy and tolerability of 3 topical pharmacotherapeutical agents (5-FU, MMC and IFN) with surgical excision and our results provided evidence that topical pharmacotherapy is as effective and well-tolerable as surgical excision in terms of tumor resolution, recurrence rate and complications in patients with OSSN.

Surgical excision has been the historical treatment of choice in OSSN, although since 1986 topical pharmacotherapeutic agents such as 5-FU, MMC and IFN have been increasingly used in the treatment of OSSN both as an alternative of the surgery and in combination^39,40^.

Surgical excision provides a shorter time to resolution, as opposed to topical pharmacotherapy where the treatment may last from few weeks to months. Excisional biopsy provides direct histopathological examination of the lesion. One of the main concerns of surgical excision is the relatively high recurrence rate ranging from 24 to 50% in the literature^41^. However, our analysis found no significant difference between topical pharmacotherapy and surgical excision regarding tumor recurrence, with a recurrence rate around 10%. Treatment outcomes were not influenced by the applied therapeutic modality regardless of tumor invasion depth or overall tumor size (surgical excision, 5-FU, IFN, MMC)^13^.

Although it is difficult to compare the recurrence rates between cases with different treatment modalities and inconsistent follow-up periods, our results show similar recurrence rates by comparing two pharmacotherapeutic agents or surgical excision with a topical agent. All the included studies showed an overall good clinical success rate regarding topical pharmacotherapy and surgical excision.

One of the main advantages of treatment with topical pharmacotherapy is that it has an effect on the entire ocular surface, including multifocal and subclinical lesions, especially in extensive disease when complete surgical excision is not possible. Topical therapeutic agents are able to penetrate into the tear drainage system and they can treat microinvasive tumors^42,43^.

We also analyzed the treatment side effects including pain, hyperemia, dry eye, keratopathy with or without limbal stem cell deficiency (LSCD) and associated systemic side effects. Earlier studies suggest that advanced surgeries (extensive limbal corneoscleral excision) can induce limbal stem cell deficiency^24^, but in the included studies there were no cases with LSCD. This could be partly explained by the limited size of the lesion chosen for complete excisional biopsy and/or the patient’s preference for topical therapy^12,13,34^. In the study conducted by Polski et al. lesions treated with surgical excision were generally smaller than those treated with topical pharmacotherapy^13^. Topical MMC and 5-FU may penetrate through the corneal epithelium and inhibit DNA and RNA synthesis in the epithelial cells. Although studies included in our meta-analysis did not report any cases of LSCD, a few case reports observed this iatrogenic complication during topical pharmacotherapy^44,45^. Both surgical and pharmacological therapy induce some side effects, but they do not remarkably influence the treatment outcomes. IFN had significantly fewer side effects than MMC, but treatment with MMC was shorter in time, IFN was found to be better tolerated than MMC^37^.

Due to similar clinical success and recurrence rates in all the therapeutic modalities, the chosen treatment can be individualized based on both physician and patient preference. One systematic review suggested the use of IFN over MMC and 5-FU due to a better tolerability^40^. Another review suggested the best therapeutic choice of topical pharmacotherapy agents being 5-FU based on storage requirements, side effect profile and treatment costs. If cost is not taken into consideration, IFN might be the best option based on the aforementioned parameters^46^.

Perilesional anti-vascular endothelial growth factors, such as bevacizumab and ranibizumab have been shown to decrease the size and vascularity of OSSN^47,48^. However, their place in the treatment of OSSN, dose and concentration remain controversial. Krilis et al. used topical retinoic acid (0.01%) with topical IFN and reported a complete resolution of OSSN in 97.75% and a long-term recurrence rate of 2.25%^49^. Topical cidofovir (2.5 mg/mL) was reported to be effective for therapy-refractive OSSN as a second-line treatment due to the anti-viral and possible anti-tumor activity^50^. In cases with corneoscleral invasion or poor response to prior surgery and/or topical pharmacotherapy, plaque brachytherapy and proton beam radiation therapy are additional treatment modalities^51,52^.

Our meta-analysis had some limitations including small sample size, the retrospective nature of the studies included and the lack of randomized controlled trials (RCTs). The number of patients varied among studies in the different treatment groups. The overall quality of evidence (GRADE) is low (Supplemental Multimedia Component 19). The four studies that provided data to the analysis carried a moderate risk of bias. The length of follow-up period after treatment was inconsistent among studies. Clinical success and recurrence were not clearly defined in one study^35^, and one study did not determine the recurrence^34^. However, it should be noted that in the study conducted by Nanji et al. only successfully treated patients were included^12^.

Implications for practice: Topical pharmacotherapy is as effective as surgical excision in terms of tumor resolution in patients with OSSN. There was no difference in recurrence rate of OSSN between topical pharmacotherapy and surgical excision highlighting similar long-term efficacy of both treatment options in OSSN.

Implication for science: Further RCTs and interventional studies are needed to compare the efficacy and safety of each topical pharmacotherapy and surgical excision in clearly defined disease groups. When designing an RCT, exact follow-up periods and standardized methods for evaluating side effects are also required.

## Conclusion

Based on our results topical pharmacotherapy may be as effective as surgical excision in terms of tumor resolution in patients with OSSN. There was no difference in recurrence rate of OSSN between topical pharmacotherapy and surgical excision indicating similar long-term efficacy of both treatment options in OSSN. Our results underline the urgent need for future randomized studies in the field that is lacking at present.

## Supplementary Information

Supplementary Information 1.

Supplementary Information 2.

## Data Availability

The data that support the findings of this study are available from the corresponding author upon reasonable request.
