# Supplementary material for: Topical pharmacotherapy for ocular surface squamous neoplasia: systematic review and meta-analysis
Source: Sci Rep. 2022 Aug 20;12:14221. doi: 10.1038/s41598-022-18545-6 (PMC9392743; doi:10.1038/s41598-022-18545-6)
Supplement: Supplementary file 2 — Supplementary Information 2. [file 41598_2022_18545_MOESM2_ESM.pdf]

Risk Of Bias Assessment

Supplemental Multimedia Component 1. Risk Of Bias Assessment - Total

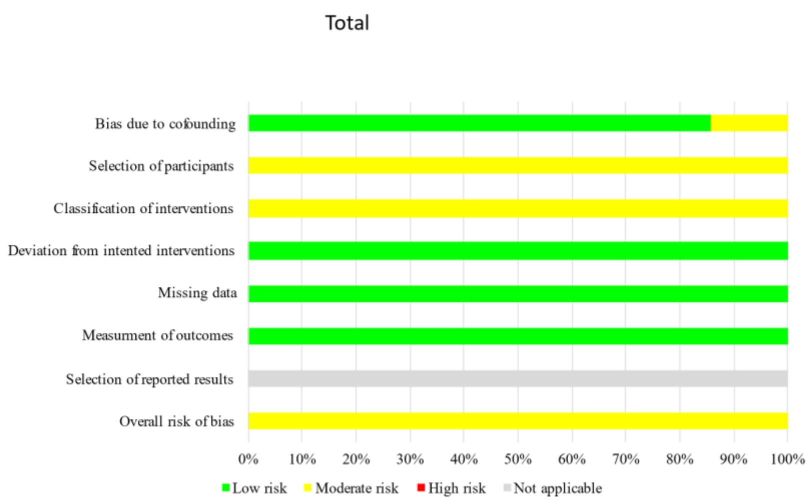

Supplemental Multimedia Component 2. Risk Of Bias Assessment – Resolution – Surgical excision vs. Topical Pharmacotherapy

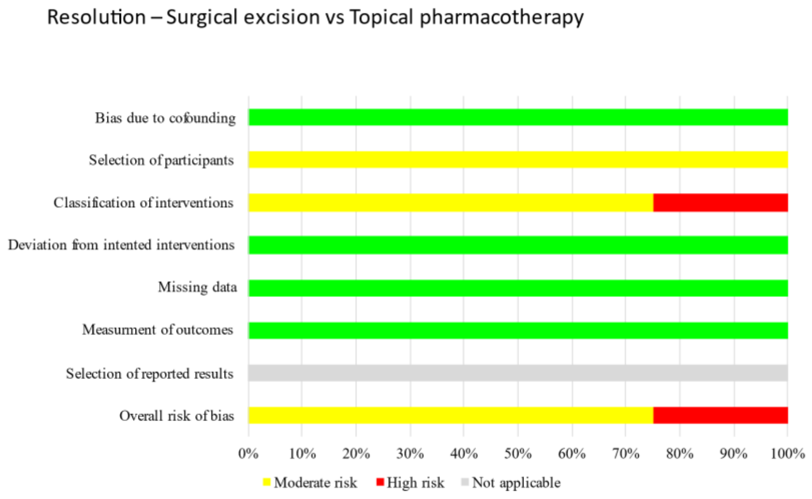

Supplemental Multimedia Component 3. Risk Of Bias Assessment – Resolution – IFN vs. 5-FU

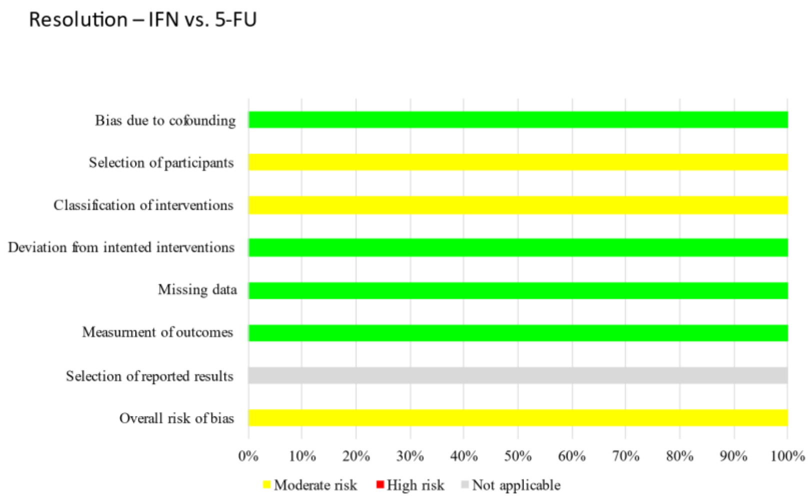

Supplemental Multimedia Component 4. Risk Of Bias Assessment – Resolution – IFN vs. MMC

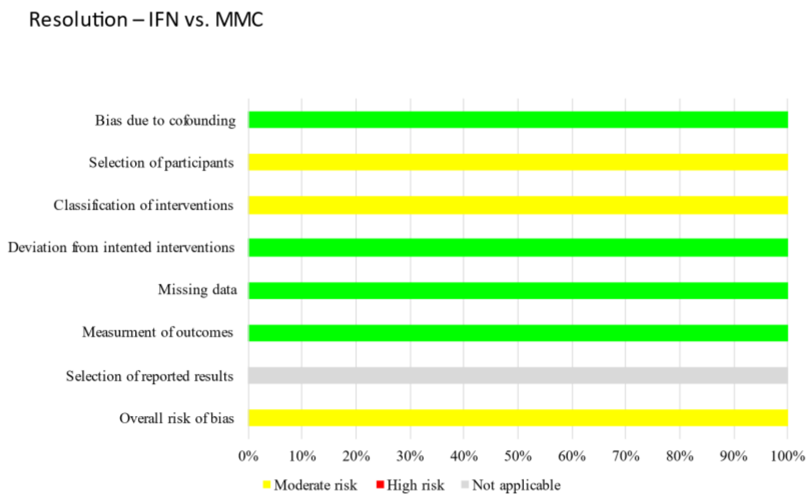

Supplemental Multimedia Component 5. Risk Of Bias Assessment – Recurrence – Surgical excision vs. Topical Pharmacotherapy

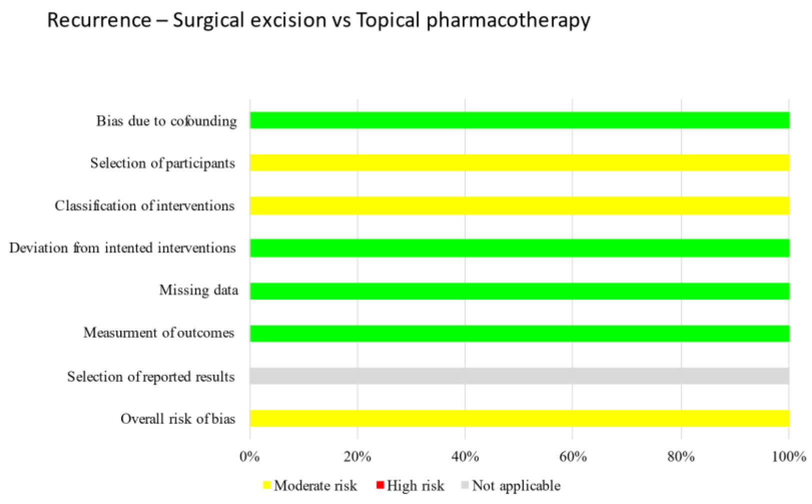

Supplemental Multimedia Component 6. Risk Of Bias Assessment – Recurrence – IFN vs. 5-FU

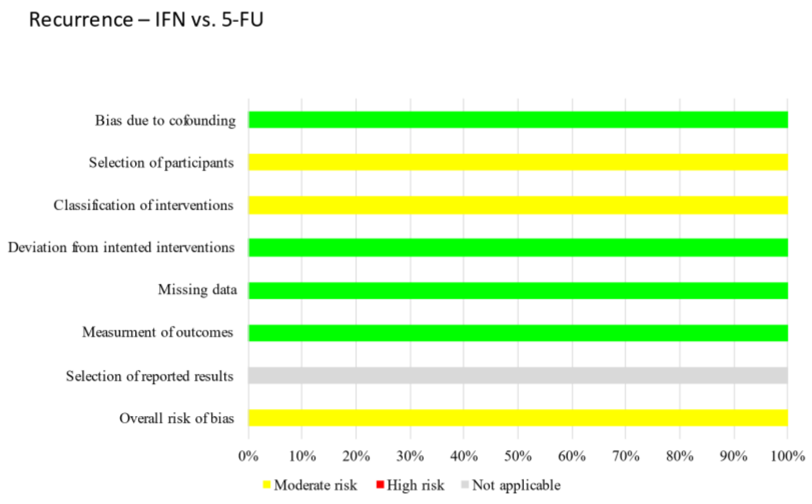

Supplemental Multimedia Component 7. Risk Of Bias Assessment – Recurrence – IFN vs. MMC

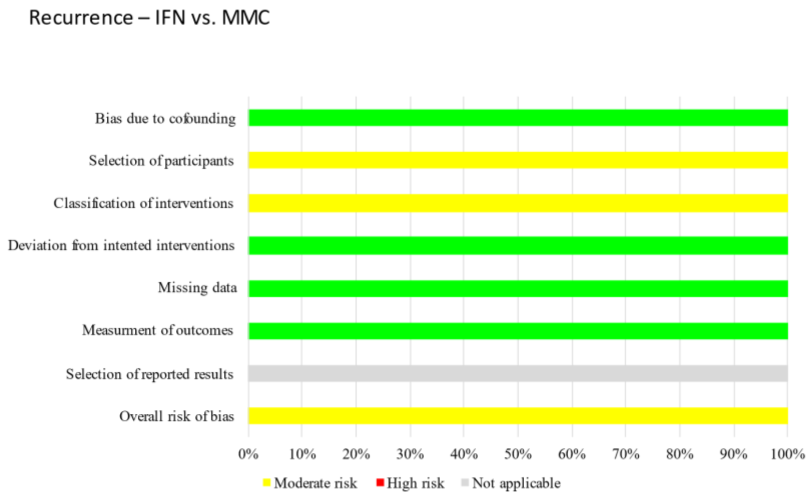

Supplemental Multimedia Component 8. Risk Of Bias Assessment – Complications Pain – Surgical excision vs. Topical Pharmacotherapy

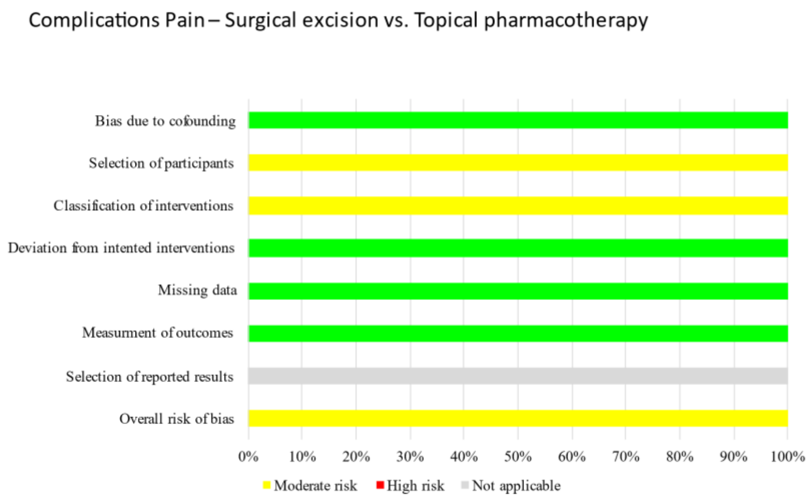

Supplemental Multimedia Component 9. Risk Of Bias Assessment – Complications Hyperaemia – Surgical excision vs. Topical Pharmacotherapy

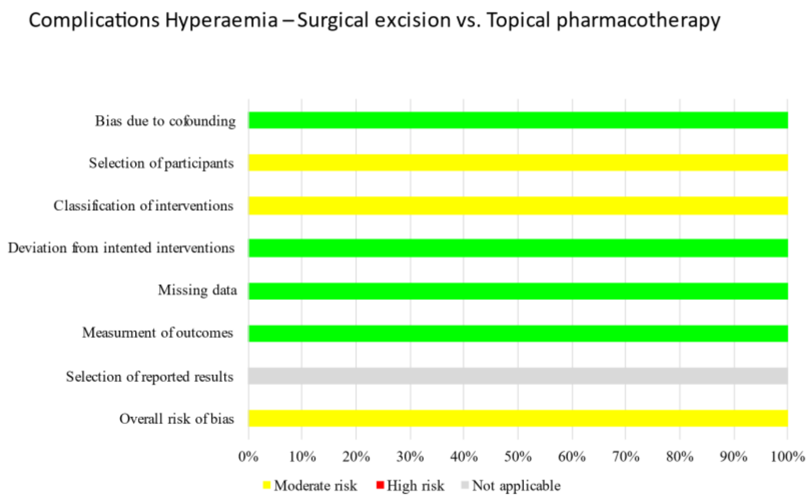

Supplemental Multimedia Component 10. Risk Of Bias Assessment – Complications Dry Eye – Surgical excision vs. Topical Pharmacotherapy

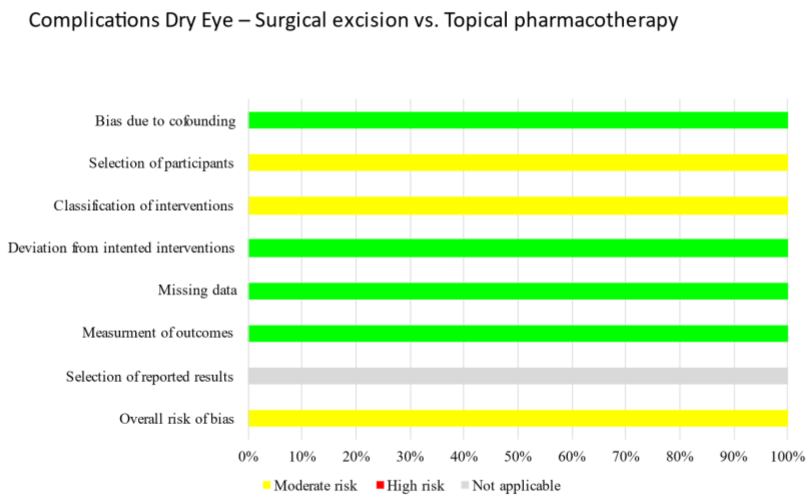

Supplemental Multimedia Component 11. Risk Of Bias Assessment – Complications Systemic Side effects – Surgical excision vs. Topical Pharmacotherapy

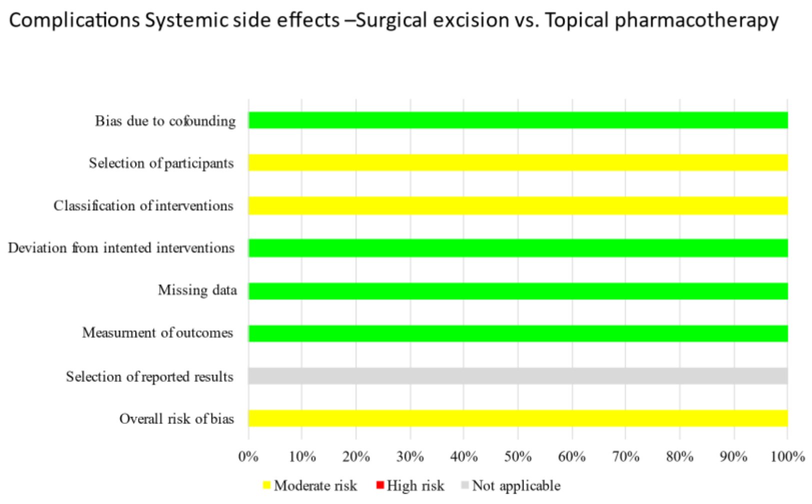

Supplemental Multimedia Component 12. Risk Of Bias Assessment – Complications Hyperaemia – IFN vs. MMC

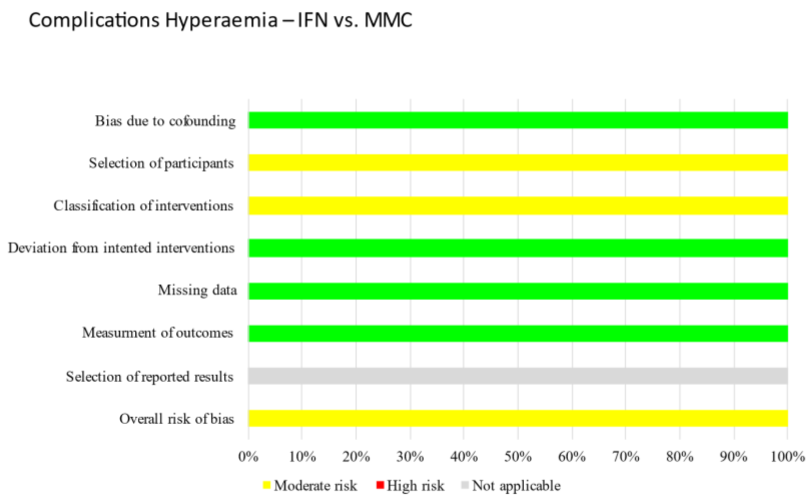

Supplemental Multimedia Component 13. Risk Of Bias Assessment – Complications Dry Eye – IFN vs. MMC

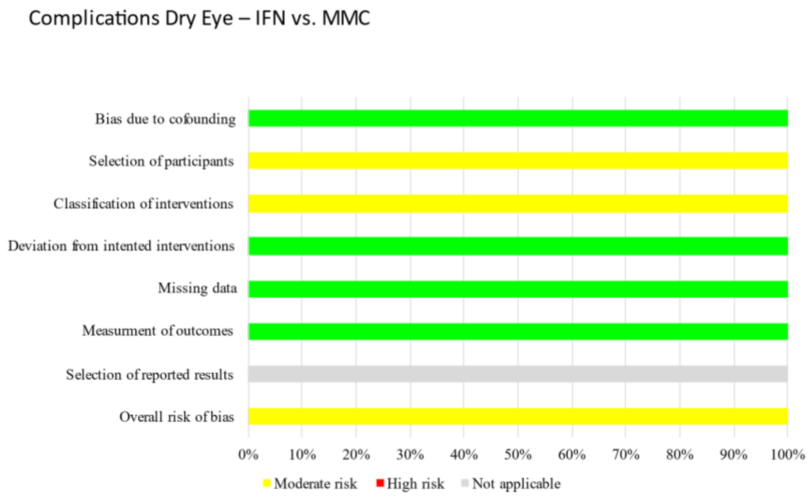

Supplemental Multimedia Component 14. Risk Of Bias Assessment – Complications Keratophaty – IFN vs. MMC

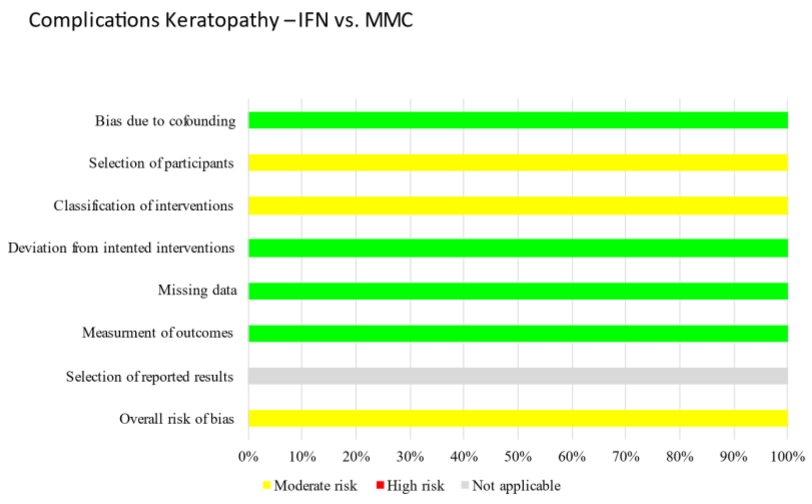

Supplemental Multimedia Component 15. Risk Of Bias Assessment – Complications Pain – IFN vs 5-FU

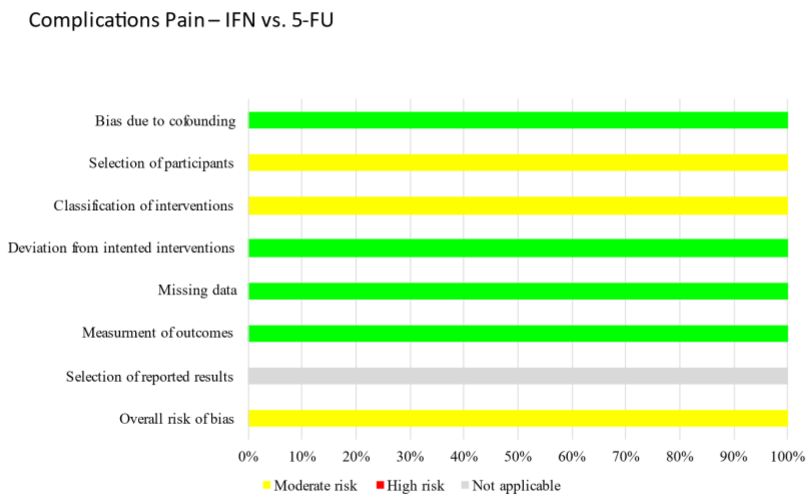

Supplemental Multimedia Component 16. Risk Of Bias Assessment – Complications Hyperaemia – IFN vs. 5-FU

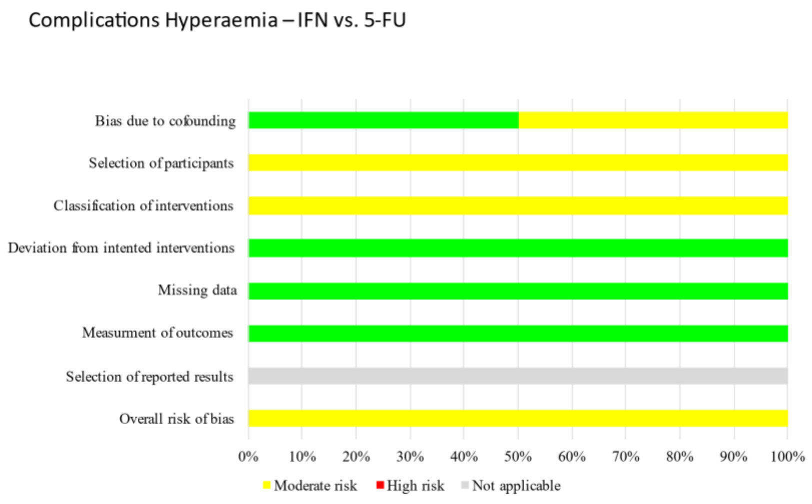

Supplemental Multimedia Component 17. Risk Of Bias Assessment – Complications Dry Eye – IFN vs. 5-FU

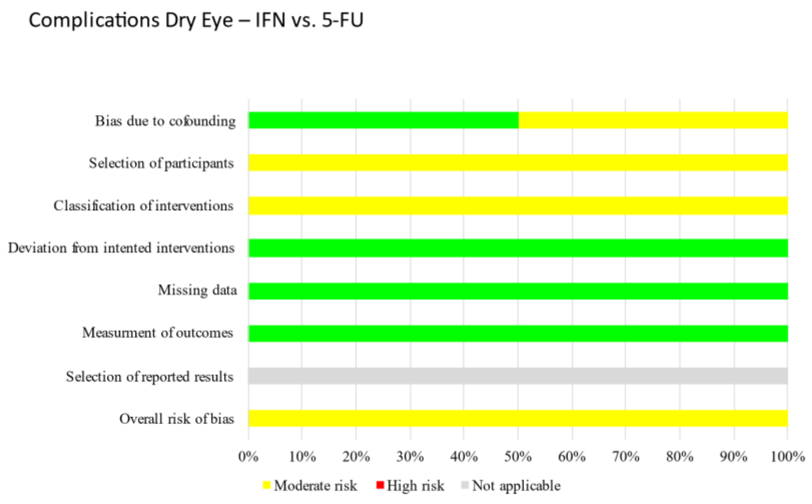

Supplemental Multimedia Component 18. Risk Of Bias Assessment – Complications Keratopathy – IFN vs. 5-FU

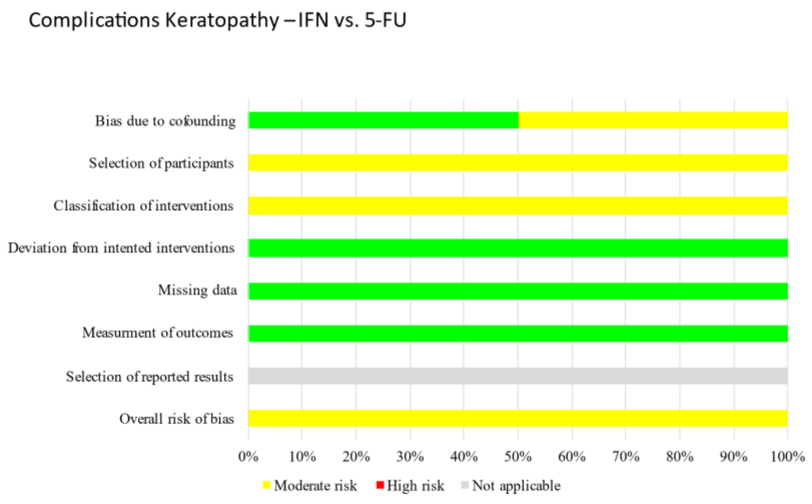

Supplemental Multimedia Component 19. GRADE

Question: Topical pharmacotherapy compared to Surgical excision for OSSN

Certainty assessment							№ of patients		Effect		Certainty	Importance
№ of studies	Study design	Risk of bias	Inconsistency	Indirectness	Imprecision	Other considerations	Topical pharmacotherapy	Surgical excision	Relative (95% CI)	Absolute (95% CI)		
**Resolution**												
4	observational studies	serious[a]	not serious	not serious	serious[b]	none	77/79 (97.5%)	79/80 (98.8%)	**OR 0.79** (0.13 to 4.74)	**3 fewer per 1 000** (from 76 fewer to 10 more)	⨁◯◯◯ Very low	CRITICAL
**Recurrence**												
4	observational studies	serious[c]	not serious	not serious	serious[b]	none	4/79 (5.1%)	6/78 (7.7%)	**OR 0.75** (0.21 to 2.61)	**18 fewer per 1 000** (from 60 fewer to 102 more)	⨁◯◯◯ Very low	CRITICAL

CI: confidence interval; **OR:** odds ratio

Explanations

a. Because all studies are observational

b. low number of events, width CI

c. Because all studies are observational
